# Ten simple rules for writing a response to reviewers

**DOI:** 10.1371/journal.pcbi.1005730

**Published:** 2017-10-12

**Authors:** William Stafford Noble

**Affiliations:** Departments of Genome Sciences and Computer Science and Engineering, University of Washington, Seattle, Washington, United States of America

You recently submitted your first manuscript for publication, and you were pleased when the editor decided to send the manuscript out for peer review. Now you have gotten the reviews back, and the editor has asked you to revise your manuscript in light of the reviewers' comments. How should you tackle this task?

Ideally, the reviewing process can significantly improve your manuscript by allowing you to take into account the advice of multiple experts in your field. Indeed, empirical evidence suggests that papers that have undergone multiple rounds of peer review fare better in terms of citation counts than papers that are quickly accepted [1]. However, in practice, the review process can be emotionally charged as you grapple with comments that may seem to you to be ill-informed, biased, or otherwise problematic.

A well-crafted "response to reviewers" document is a critical part of your response. This document is submitted alongside your revised manuscript, summarizing the changes that you made in response to the critiques. Too frequently, authors focus on revising the manuscript itself and spend too little time making the response document clear and compelling. The result can be misunderstandings between the reviewers and the authors and ultimately, the possible rejection of a high-quality manuscript. Following are 10 simple rules that can help in formulating an effective response to reviewers.

## Rule 1: Provide an overview, then quote the full set of reviews

The response letter will typically begin with a summary of changes, pointing out new data and new analyses performed in response to the most essential criticisms of all the reviewers. Note that, at your discretion, the response may include figures and tables that are for the reviewers' benefit but will not go into the manuscript or supplement. These additional results can be mentioned in your Introduction. If a criticism is raised by multiple reviewers, this can also be pointed out in the summary. Thereafter, the response letter should contain the complete set of reviews with your responses interleaved.

## Rule 2: Be polite and respectful of all reviewers

Even if you are convinced that the reviewer lacks intellectual capacity, it is certainly not in your interest to convey this impression to the reviewer. Keep in mind that if the reviewer failed to understand something, the fault likely lies, at least in part, with you for not making the point clear enough. If the reviewer does not seem to be an expert in the area, remember that this level of expertise (or lack thereof) may be representative of many readers of the journal. Your goal is to make the work clear and accessible to all readers, not just to experts.

Sometimes you will need to work to understand a particular critique. In some cases, the question the reviewer asks reveals a deeper misunderstanding about the overall study or some of the assumptions therein. When specific comments seem off-base, and especially when a single reviewer has many such comments, this may be because the manuscript does not sufficiently explain the hypothesis it aims to address.

In some cases, you may believe that the reviewer is vengeful or is a competitor who has an ulterior motive to delay the manuscript. In such situations, you should not directly confront the reviewer in your response but instead communicate your concerns to the editors in a separate letter.

In rare cases, you may feel that a reviewer's critiques are simply discourteous. In such situations, it is important to remember that miscommunications are possible. Regardless, a rude critique does not justify a rude response from you, especially because your primary goal is to publish your scientific results.

## Rule 3: Accept the blame

If the reviewer failed to understand something, apologize for not making it clear. Even if you are convinced that the text is already clear (i.e., the reviewer simply missed it), consider revising the text and quoting the revised text in your response. In general, even if the requested change seems unnecessary, it is usually better to go ahead and revise with the goal of showing the reviewer that they were listened to and understood.

## Rule 4: Make the response self-contained

When you make changes to the text or to figures, quote the changes directly in the response. If possible, you can refer to the specific line number where the changes were applied, though you should be sure to specify whether you refer to the line numbers from the original or the revised manuscript. A self-contained response letter makes it easier for the reviewer to understand exactly what you did without having to flip back and forth between your manuscript and the response. Furthermore, by making your response self-contained, you reduce the likelihood that the reviewer will read the full manuscript and find new things to complain about. The only exception to this rule is when a large chunk of modified text (e.g., a new section) is too long to quote. Such changes can simply be alluded to explicitly (e.g., giving the title of the new section) in the response.

## Rule 5: Respond to every point raised by the reviewer

A frequent complaint from reviewers is that the authors failed to respond at all to several points raised in the review. In some cases, the reviewer may disagree with your response, but you should not try to avoid a difficult point by simply ignoring it.

Often, reviews will be organized into bullet points, but the reviewer may raise 2 separate issues within 1 bullet. In such situations, be sure to respond explicitly to both critiques. It is fine for you to interleave your responses in such a way that you break up 1 bullet with multiple responses. It is usually better to do this than to try to respond to multiple points in 1 block of text.

## Rule 6: Use typography to help the reviewer navigate your response

Use changes of typeface, color, and indenting to discriminate between 3 different elements: the review itself, your responses to the review, and changes that you have made to the manuscript. You can explain these typographical conventions in the introduction to your response.

## Rule 7: Whenever possible, begin your response to each comment with a direct answer to the point being raised

You can provide background information, but you should do so after giving your primary response. Provide a “yes” or “no” answer whenever possible. When the reviewer is correct, state so in your response. Your goal is to show the reviewer that you took their comments seriously, and you should quickly convey what you did in response to their critique.

## Rule 8: When possible, do what the reviewer asks

In general, you should avoid giving the impression that you couldn't be bothered to carry out the additional experiments or analyses that the reviewer asks for. Even in cases in which you believe the reviewer has requested an analysis that you don’t find informative, or is otherwise flawed, you will often be in a stronger position if you do what the reviewer asked, report the results in your response, and then explain why you believe the results do not belong in your manuscript.

In some cases, if the reviewer makes detailed or very insightful suggestions that get incorporated into the revised manuscript, it may be appropriate to add to the Acknowledgments section an explicit "thank you" to the reviewer. Indeed, many authors routinely include an acknowledgment of the reviewers in all of their publications. Note, however, that some journals (including *PLOS Computational Biology*) do not allow reviewer acknowledgments.

Sometimes reviewers simply ask for too much. It is certainly acceptable to say that the requests go beyond what you perceive to be the scope of the current work. However, it is also important to recognize that the scope of a given manuscript is often difficult to define precisely. If the reviewer asks for 10 things, and you say that 9 out of 10 of them fall outside the scope of your work, then you are not likely to satisfy the reviewer. In such a situation, you may need to do a few things that you think fall outside the scope of your original work.

Occasionally, it may be necessary to fall back on the discretion of the editor. For example, editors often ask that authors shorten their manuscripts, whereas reviewers often ask for additional details, experiments, or analyses. If, for example, a reviewer asks you to move some content from the supplement to the main manuscript, you may want to say that you are willing to do so if the editor concurs.

## Rule 9: Be clear about what changed relative to the previous version

When you make a change in response to a reviewer's comment, it can sometimes be difficult to convey to the reviewer exactly what that change consisted of. A common error is for an author to respond to a reviewer's comment by saying, "This point is addressed in the manuscript in the following way…" This response fails to make clear whether the author is simply pointing out text that was already present in the previous version of the manuscript, or the author is describing changes that have been incorporated into the new version. In your response, refer explicitly to the previous and revised versions of your manuscript and explain what changes have been made.

## Rule 10: If necessary, write the response twice

Your initial draft of the “response to reviewers” document may aim to analyze what the reviewer meant while considering different avenues of response and the cost–benefit tradeoff of performing additional experiments. This document can be helpful to you and your coauthors as you decide how to formulate a final response document. The initial document can also be a place to vent your frustration with what you perceive to be unfair or rude reviews. After writing this initial draft, you can begin writing a completely separate document that contains what you actually want the reviewers to see. In practice, it is often helpful to write the "venting" version of the response first, wait a while, and then begin working on the "real" response several days later, perhaps after you have done some of the work to address the critiques raised by the reviewer.

In addition to the "response to reviewers" letter, you may in some cases want to write a separate letter to the managing editor. In this letter, you can address issues about potential conflicts of interest. You may also want to point out when the reviewers' requests conflict with one another or with journal policies.

The process of responding to reviewer critiques can be one of the more stressful parts of the publication process. Throughout the process, it is helpful to keep in mind that, in most cases, the reviewers are well-meaning colleagues who are volunteering their time to help ensure the validity of results that are reported in the scientific literature. In nearly every case, the manuscript that comes out of the review process is improved relative to the original version.
